# Patterns of SARS-CoV-2 Testing Preferences in a National Cohort in the United States: Latent Class Analysis of a Discrete Choice Experiment

**DOI:** 10.2196/32846

**Published:** 2021-12-30

**Authors:** Rebecca Zimba, Matthew L Romo, Sarah G Kulkarni, Amanda Berry, William You, Chloe Mirzayi, Drew A Westmoreland, Angela M Parcesepe, Levi Waldron, Madhura S Rane, Shivani Kochhar, McKaylee M Robertson, Andrew R Maroko, Christian Grov, Denis Nash

**Affiliations:** 1 Institute for Implementation Science in Population Health CUNY Graduate School of Public Health & Health Policy New York, NY United States; 2 Department of Epidemiology and Biostatistics CUNY Graduate School of Public Health & Health Policy New York, NY United States; 3 Department of Maternal and Child Health Gillings School of Public Health University of North Carolina Chapel Hill, NC United States; 4 Carolina Population Center University of North Carolina at Chapel Hill Chapel Hill, NC United States; 5 Department of Environmental, Occupational, and Geospatial Health Sciences CUNY Graduate School of Public Health & Health Policy New York, NY United States; 6 Department of Community Health and Social Sciences CUNY Graduate School of Public Health & Health Policy New York, NY United States

**Keywords:** SARS-CoV-2, testing, discrete choice experiment, latent class analysis, COVID-19, pattern, trend, preference, cohort, United States, discrete choice, diagnostic, transmission, vaccine, uptake, public health

## Abstract

**Background:**

Inadequate screening and diagnostic testing in the United States throughout the first several months of the COVID-19 pandemic led to undetected cases transmitting disease in the community and an underestimation of cases. Though testing supply has increased, maintaining testing uptake remains a public health priority in the efforts to control community transmission considering the availability of vaccinations and threats from variants.

**Objective:**

This study aimed to identify patterns of preferences for SARS-CoV-2 screening and diagnostic testing prior to widespread vaccine availability and uptake.

**Methods:**

We conducted a discrete choice experiment (DCE) among participants in the national, prospective CHASING COVID (Communities, Households, and SARS-CoV-2 Epidemiology) Cohort Study from July 30 to September 8, 2020. The DCE elicited preferences for SARS-CoV-2 test type, specimen type, testing venue, and result turnaround time. We used latent class multinomial logit to identify distinct patterns of preferences related to testing as measured by attribute-level part-worth utilities and conducted a simulation based on the utility estimates to predict testing uptake if additional testing scenarios were offered.

**Results:**

Of the 5098 invited cohort participants, 4793 (94.0%) completed the DCE. Five distinct patterns of SARS-CoV-2 testing emerged. Noninvasive home testers (n=920, 19.2% of participants) were most influenced by specimen type and favored less invasive specimen collection methods, with saliva being most preferred; this group was the least likely to opt out of testing. Fast-track testers (n=1235, 25.8%) were most influenced by result turnaround time and favored immediate and same-day turnaround time. Among dual testers (n=889, 18.5%), test type was the most important attribute, and preference was given to both antibody and viral tests. Noninvasive dual testers (n=1578, 32.9%) were most strongly influenced by specimen type and test type, preferring saliva and cheek swab specimens and both antibody and viral tests. Among hesitant home testers (n=171, 3.6%), the venue was the most important attribute; notably, this group was the most likely to opt out of testing. In addition to variability in preferences for testing features, heterogeneity was observed in the distribution of certain demographic characteristics (age, race/ethnicity, education, and employment), history of SARS-CoV-2 testing, COVID-19 diagnosis, and concern about the pandemic. Simulation models predicted that testing uptake would increase from 81.6% (with a status quo scenario of polymerase chain reaction by nasal swab in a provider’s office and a turnaround time of several days) to 98.1% by offering additional scenarios using less invasive specimens, both viral and antibody tests from a single specimen, faster turnaround time, and at-home testing.

**Conclusions:**

We identified substantial differences in preferences for SARS-CoV-2 testing and found that offering additional testing options would likely increase testing uptake in line with public health goals. Additional studies may be warranted to understand if preferences for testing have changed since the availability and widespread uptake of vaccines.

## Introduction

Screening and diagnostic testing for SARS-CoV-2 infection is a critical tool in the public health response to the COVID-19 pandemic, as early detection allows for the implementation of isolation and quarantine measures to reduce community transmission [1]. Negative tests are often required for work, school, and leisure activities. The importance of testing has been well demonstrated globally, such as in South Korea, where a “test, trace, isolate” strategy was largely credited for rapidly controlling transmission in spring 2020 [2]. Unfortunately, insufficient SARS-CoV-2 testing in the United States throughout the first several months of the pandemic led to both undetected cases transmitting disease in the community and an underestimation of the burden of COVID-19 [3]. Though the SARS-CoV-2 diagnostic testing supply has increased, maintaining testing uptake remains a major US public health priority in the efforts to control community transmission in the current pandemic phase of vaccinations and variants [4-6]. Currently, the US Centers for Disease Control and Prevention recommends diagnostic testing for individuals with symptoms of COVID-19 and unvaccinated individuals in close contact with a confirmed or suspected COVID-19 case; they also recommend screening tests for unvaccinated people, for example, for work, school, or travel [7-9]. Hereafter, we define SARS-CoV-2 testing as including both screening and diagnostic testing.

Individuals’ preferences about testing, specifically about the test itself or the service model that delivers the test, are important to consider in determining strategies to increase and maintain the uptake of SARS-CoV-2 testing in the vaccine era. In other contexts, individual preferences about a health-related product or service have been shown to be predictive of adoption of health-related behaviors [10]. Discrete choice experiments (DCEs), which are surveys that elicit stated preferences to identify trade-offs that a person makes with a product or service, have emerged as a tool to understand patient preferences and barriers to health care engagement [11], and are increasingly being used to inform patient-centered health care [12,13]. We previously conducted a DCE to understand SARS-CoV-2 testing preferences and found strong preferences for both viral and antibody testing, less invasive specimen collection, and rapid result turnaround time [14]. However, as observed in DCEs on other topics, patient preferences are often heterogeneous, and there may be distinct patterns of preferences within a population [15].

If indeed preferences are relevant to SARS-CoV-2 testing uptake and different patterns of preferences exist, these patterns may also be characterized by distinct demographic profiles. Previous work has documented demographic disparities in SARS-CoV-2 testing uptake [16-19]. For example, among individuals receiving care at US Department of Veterans Affairs sites, overall SARS-CoV-2 testing rates within the Veterans Affairs system were lowest among non-Hispanic White individuals, especially among those who were male and those who lived in rural settings; however, testing rates per positive case were lowest among non-Hispanic Black and Hispanic individuals [17]. Patterns of preferences may also differ based on experience with a product or service, as observed with the frequency of past testing in the preferences for HIV self-testing [20]. Concern or perceived risk is another component involved in making decisions about health, and since it is not uniformly distributed, it may also differ by patterns of preferences [21].

We hypothesized that discernable patterns of SARS-CoV-2 testing preferences would emerge and that individuals in these patterns would have distinct demographic profiles, SARS-CoV-2 testing history, and concern about infection. Identifying and characterizing heterogenous testing preferences could facilitate the design and implementation of an array of services and ultimately enhance testing uptake and engagement.

## Methods

### Recruitment and Study Ethics

The survey design of our DCE has been previously described [14], but here we provide a summary. Participants enrolled in the CHASING COVID (Communities, Households, and SARS-CoV-2 Epidemiology) Cohort Study [22] who completed a routine follow-up assessment from July 30 through September 8, 2020, were invited to complete the DCE via a unique survey link at the end of the follow-up assessment. A US $5 Amazon gift card incentive was offered to participants completing the DCE. All study procedures were approved by the City University of New York (CUNY) Graduate School of Public Health and Health Policy Institutional Review Board.

### DCE Design

The DCE was designed and implemented using Lighthouse Studio 9.8.1 (Sawtooth Software). Prior to the main DCE tasks, participants who agreed to complete the DCE were presented with a sample task related to ice cream preferences to help demonstrate the method and orient them to the DCE format. Each participant was then presented with 5 choice tasks with illustrations where they were asked to indicate which of 2 different SARS-CoV-2 testing scenarios was preferable or if neither was acceptable, imagining that “...the number of people hospitalized or dying from the coronavirus in your community was increasing” (see Multimedia Appendix 1 for a sample choice task). Specific testing attributes and levels examined are described in Multimedia Appendix 2. These attributes and levels were based on the current options for SARS-CoV-2 testing in the United States at the time of study design, as well as aspirational features hypothesized by the study investigators to be relevant to individual preferences. The combination and order of attribute levels presented to each participant were randomized for a balanced and orthogonal design using Sawtooth’s Balanced Overlap method [23,24]. However, we did constrain the combination of certain levels to reflect real-world possibilities. For example, we did not allow the nasopharyngeal (NP) swab specimen type level to be combined with either of the at-home specimen collection venue levels. The survey was tested internally by study team members prior to deployment.

### Latent Class Analysis

For the unsegmented analysis reported previously [14], we estimated individual-level part-worth utilities for each attribute level and relative importance for each attribute using a hierarchical Bayesian multinomial logit (MNL) model, which iterates through the upper aggregate level of the hierarchy and the lower individual level of the hierarchy until convergence [25,26]. For this analysis, however, we used a latent class MNL model to identify different segments of respondents based on their response patterns. Latent class MNL estimation first selects random estimates of each segment’s utility values, then uses those values to fit each participant’s data and estimate the relative probability of each respondent belonging to each class [27]. Next, using probabilities as weights, logit weights are re-estimated for each pattern and log-likelihoods are accumulated across all classes. This process repeats until reaching the convergence limit. We estimated individual-level utilities as the weighted average of the group utilities weighted by each participant’s likelihood of belonging to each group, and zero-centered the utilities using effects coding, so that the reference level is the negative sum of the preferences of the other levels within each attribute [28-31]. Compared to aggregate logit, the effect of the independence of irrelevant alternatives assumption is reduced in latent class MNL [28]. We calculated relative importance at the individual level as the range of utilities within an attribute over the sum of the ranges of utilities of all attributes, and then calculated the mean and 95% CI for each segment as the mean ± 1.96 × SE. We calculated the mean of the weighted utilities by segment, and 95% CIs in the same manner as previously described [29,32].

We ran the latent class analysis with 2 to 10 classes, 5 replications per class, and 100 iterations per replication to facilitate convergence. We used the Akaike information criterion, Bayesian information criterion, and log-likelihood to inform best model selection. In addition, we sought a segmentation model that balanced statistical fit with interpretability and reasonably sized groups. We ran the latent class analysis with multiple starting seeds to facilitate finding the globally best-fit solution.

### Respondent Quality

To assess respondent quality, we computed DCE exercise completion time statistics and examined straightlining behavior (always picking the left-hand alternative or the right-hand alternative) [33]. We reran the latent class analysis excluding participants with a combination of straightlining behavior, or completion times in the 5th or 10th percentile of all participants, to determine whether these participants affected the final model. All latent class analyses were done using Lighthouse Studio 9.8.1.

### Simulation

We then extrapolated the 2-alternative choice task to a multiscenario simulation to estimate preferences for 5 testing approaches, summarized in Table 1, using the individual-level part-worth utility estimates from the latent class MNL analysis and stratifying by the latent class segments. Our simulations included the following:

- Scenarios 1 and 2: “standard testing” scenarios were based on major health departments’ testing programs in fall 2020—viral test (NP swab) and a result turnaround time of 48 hours. We included two unique scenarios to cover two variations in venue [34,35]—drive-through testing site and walk-in community testing site. Result turnaround time was based on reports from major health departments (eg, >85% of results within 2 days in California) [35].
- Scenario 3: “less invasive testing” was based on some jurisdictions offering less invasive specimen collection, such as saliva [34]—viral test, saliva specimen, walk-in community testing site, and a result turnaround time of 48 hours.
- Scenario 4: “dual testing” consisted of both viral and antibody testing and would necessitate a finger prick [36]—viral and antibody test, finger prick specimen, walk-in community testing site, and a result turnaround time of 48 hours.
- Scenario 5: “at-home testing” was based on a commercially available at-home testing kit [37]—viral test, shallow nasal swab, home collection, receiving and returning the kit in the mail, and a result turnaround time within 5 days, as additional time would be required for mailing the specimen.

We conducted two sets of simulations to predict testing uptake: (1) the 2 standard testing scenarios, with a “no test” option to capture the proportion of participants in each class who would opt out of testing altogether, given the choices, and (2) the 2 standard test scenarios as well as the less invasive, dual testing, and at-home testing scenarios, including a “no test” option. Predicted uptake for the 3 total options for the first simulation and 6 total options for the second simulation were generated using the Randomized First Choice (RFC) method with utilities from the latent class MNL as inputs [25,38,39]. The RFC approach assumes that participants would choose the testing scenario with the highest total utility summed across attributes using each participant’s own individual estimated utilities, with some perturbation around the utilities to account for test scenario similarities and reduce the independence of an irrelevant alternatives problem. The simulator performs thousands of simulated draws per participant, then computes the proportion of participants who would choose each testing scenario based on its total utility. The simulations were done using Lighthouse Studio 9.8.1.

**Table 1:** Testing approaches used in the simulations.

Testing scenario		Test	Specimen type	Venue	Result turnaround time	Included in simulation 1	Included in simulation 2
1.	Standard testing, drive-through	PCR^a^	NP^b^ swab	Drive-through community testing site	48 hours	✓^c^	✓
2.	Standard testing, walk-in	PCR	NP swab	Walk-in community testing site	48 hours	✓	✓
3.	Less invasive testing	PCR	Spit sample	Walk-in community testing site	48 hours		✓
4.	Dual testing	PCR and serology	Finger prick	Walk-in community testing site	48 hours		✓
5.	At-home testing	PCR	Shallow nasal swab	Home collection, receiving and returning the kit via mail	Within 5 days		✓
6.	None	N/A	N/A	N/A	N/A	✓	✓

^a^PCR: polymerase chain reaction.

^b^NP: nasopharyngeal.

^c^✓: Check marks indicate whether the testing scenario was included in each simulation.

### Additional Measures

Other measures of interest were merged from participants’ responses from the CHASING COVID Cohort Study [22] baseline interview (age, gender, race/ethnicity, education, region, urbanicity, comorbidities) and a combination of baseline, visit 1, and visit 2 follow-up interviews (employment, concern about infection, previous SARS-CoV-2 testing) [40] (see Multimedia Appendix 3 for details on how the variables were defined).

We computed descriptive statistics (frequencies and proportions) for these characteristics by class and compared the distributions of these variables using Pearson chi-square tests. An alpha level of .05 was the criterion for statistical significance. The descriptive statistics and bivariate analyses were done using SAS 9.4 (SAS Institute).

## Results

### Participant Demographic Characteristics

Of the 5098 invited cohort participants, 4793 participants completed the DCE (response rate 94.0%). The median age was 39 (IQR 30-53) years, 51.5% (n=2468) were female, 62.8% (n=3009) were non-Hispanic White, 16.4% (n=788) were Hispanic, 9.2% (n=442) were non-Hispanic Black, 7.4% (n=361) were Asian or Pacific Islander, and 3.9% (n=189) were another race.

### Respondent Quality

We assessed respondent quality and reran the 5-group latent class analysis four times excluding participants who exhibited combinations of either straightlining behavior (n=392), completion times in the 5th (n=239) or 10th (n=473) percentile of all responders, or combinations of straightlining and speeding. Though there was some volatility in the part-worth utility estimates for the smallest class size in the models with exclusions, there were no qualitative differences to class sizes or relative attribute importance. Therefore, we used the model that retained all 4793 participants (see Multimedia Appendix 4 for additional details).

### Patterns of Preferences: Relative Attribute Importance and Preferences for Levels of Attributes

**Figure 1:**
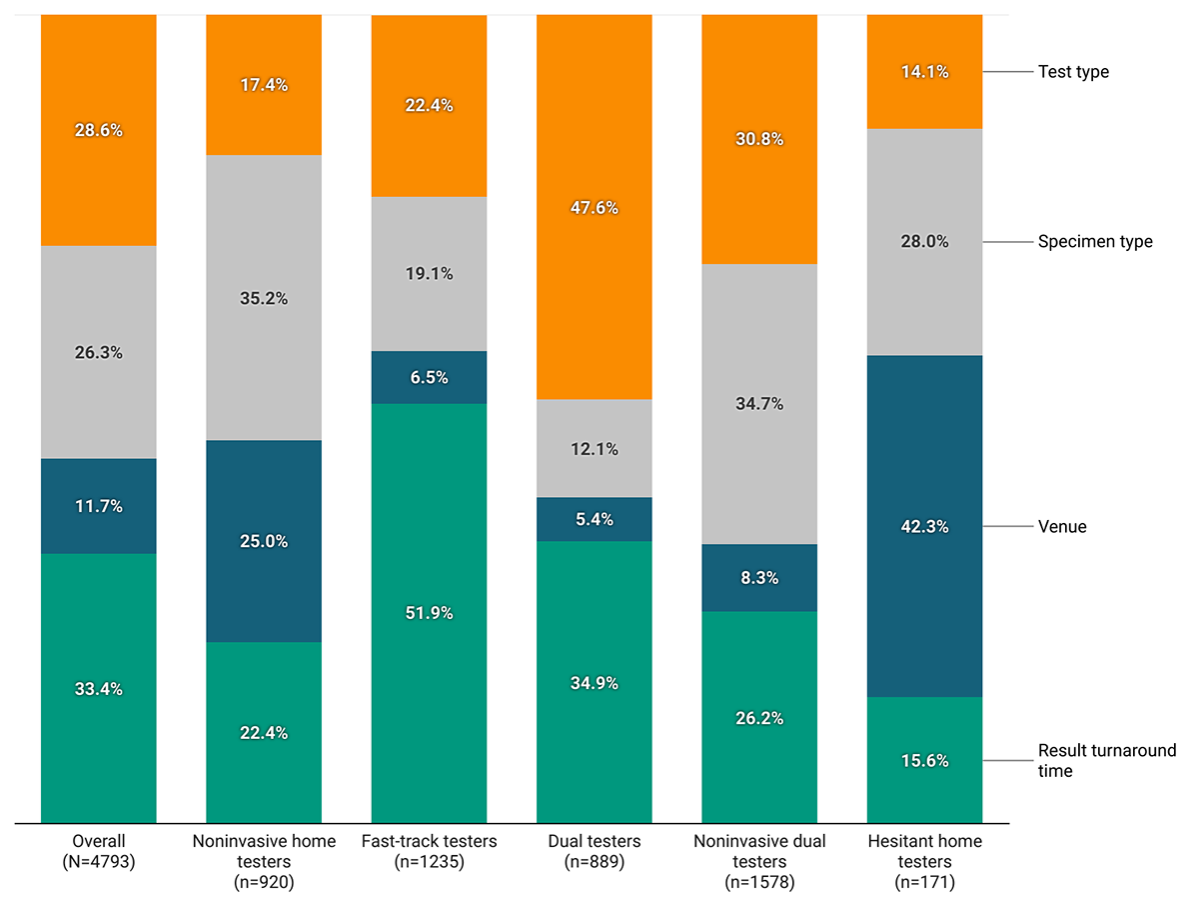
Mean relative attribute importance for SARS-CoV-2 testing by preference pattern.

Among the 4793 participants who completed the DCE, 5 distinct classes were identified balancing quantitative measures of model fit (Akaike information criterion, Bayesian information criterion, and log-likelihood) with class size and the ability to interpret the final solution. Multimedia Appendix 5 presents a summary of these criteria. Each class had a distinct profile or pattern of attribute relative importance (Figure 1) and preferences for specific levels of attributes (Table S1, Multimedia Appendix 6). We characterized the patterns based on the preferences within each class: noninvasive home testers (n=920, 19.2%), fast-track testers (n=1235, 25.8%), dual testers (n=889, 18.5%), noninvasive dual testers (n=1578, 32.9%), and hesitant home testers (n=171, 3.6%).

To elaborate, among noninvasive home testers*,* specimen type had the highest relative importance (35.2%, 95% CI 34.8%-35.5%), followed by venue (25.0%, 95% CI 24.4%-25.5%), result turnaround time (22.4%, 95% CI 22.0%-22.9%), and test type (17.4%, 95% CI 16.9%-17.9%). Participants in this pattern favored less invasive specimen types, with saliva being most preferred (utility 48.6) and NP swab and blood draw specimen types being least preferred (utilities –92.1 and –41.3, respectively). They preferred home sample collection, either returning the sample for testing by mail (utility 55.3) or to a collection site (utility 42.7), and least preferred testing at a doctor’s office or urgent care clinic (utility –41.6) or walk-in community testing site (utility –44.5). Participants in this pattern most preferred a fast turnaround time for their results (immediate, utility 42.0; same day, utility 30.4) and antibody and viral tests together (utility 39.5). The none option had a large negative utility (–299.6).

The attribute with the highest relative importance for fast-track testers was result turnaround time (51.9%, 95% CI 51.6%-52.3%), followed by test type (22.4%, 95% CI 22.1%-22.8%) and specimen type (19.1%, 95% CI 18.8%-19.4%); venue was least important (6.5%, 95% CI 6.4%-6.6%). Participants in this pattern had the most extreme range of values for relative importance. They most preferred an immediate (utility 98.6) and same-day (utility 63.8) result turnaround time and both antibody and viral tests (utility 53.0). They preferred less invasive specimen types, with a cheek swab being most preferred (utility 25.4) and NP swab and blood draw being least preferred (utilities –51.1 and –20.7, respectively). Although venue was the least important attribute for this group, among the specific options, testing at a community drive-through was most preferred (utility 10.8). Similar to noninvasive home testers*,* the fast-track testers had a large negative utility for the none option (–238.0).

Among dual testers*,* test type had the highest relative importance (47.6%, 95% CI 47.2%-47.9%), followed by result turnaround time (34.9%, 95% CI 34.7%-35.2%), with specimen type being less important (12.2%, 95% CI 11.8%-12.4%) and venue being least important (5.4%, 95% CI 5.3%-5.5%). Participants in this pattern most preferred both antibody and viral tests (utility 93.3) and fast turnaround times for results (immediate: utility 64.9; same day: utility 40.5). Less invasive specimen types were preferred, with saliva (utility 14.2) and cheek swab (utility 16.4) being most preferred, and NP swab being least preferred (utility –30.5). Regarding venue, testing at drive-through community sites was most preferred (utility 12.2). Dual testers had a large negative utility for the none opt-out choice (utility –217.4).

Among noninvasive dual testers*,* specimen type had the highest relative importance (34.7%, 95% CI 34.5%-34.8%), followed by test type (30.8%, 95% CI 30.7%-31.0%) and then result turnaround time (26.2%, 95% CI 26.0%-26.5%), with venue least important (8.3%, 95% CI 8.2%-8.4%). In this pattern, the most preferred specimen types were saliva (utility 28.1) and cheek swab (utility 37.8), and the least preferred was NP swab (utility –100.9). Both antibody and viral tests were preferred (utility 73.3), as well as fast turnaround times for results (immediate: utility 54.7; same day: utility 25.1). Regarding venue, home collection with returning the sample to a collection site was most preferred (utility 16.8). Similar to the previous three patterns, noninvasive dual testers had a large negative utility for the none opt-out choice (–226.6).

Finally, among participants in the hesitant home testers pattern, venue had the highest relative importance (42.3%, 95% CI 41.4%-43.1%) followed by specimen type (28.0%, 95% CI 27.7%-28.2%); result turnaround time (15.6%, 95% CI 14.9%-16.4%) and test type (14.1%, 95% CI 13.8%-14.5%) were similarly less important. In contrast to the other 4 patterns, hesitant home testers had a positive utility (32.5) for the none option, hence the use of “hesitant” in this pattern’s name. Participants in this pattern preferred less invasive specimens, including urine (utility 33.5), finger prick (utility 24.5), cheek swab and saliva (utilities 25.6 and 18.7, respectively), and least preferred NP swab (utility –77.7) and blood draw (utility –21.1). They most preferred home sample collection either returning the sample for testing by mail (utility 93.2) or to a collection site (utility 60.2), and least preferred testing at a walk-in community site (utility –75.9) or a doctor’s office/urgent care clinic (utility –44.6). Although test type and turnaround time were the least important attributes for hesitant home testers, participants with this pattern preferred both antibody and viral tests (utility 36.1) and fast turnaround times for results (immediate: utility 32.5; same day: utility 21.2).

### Simulated Preferences for Standard Testing, Less Invasive Testing, Dual Testing, and At-Home Testing

**Figure 2:**
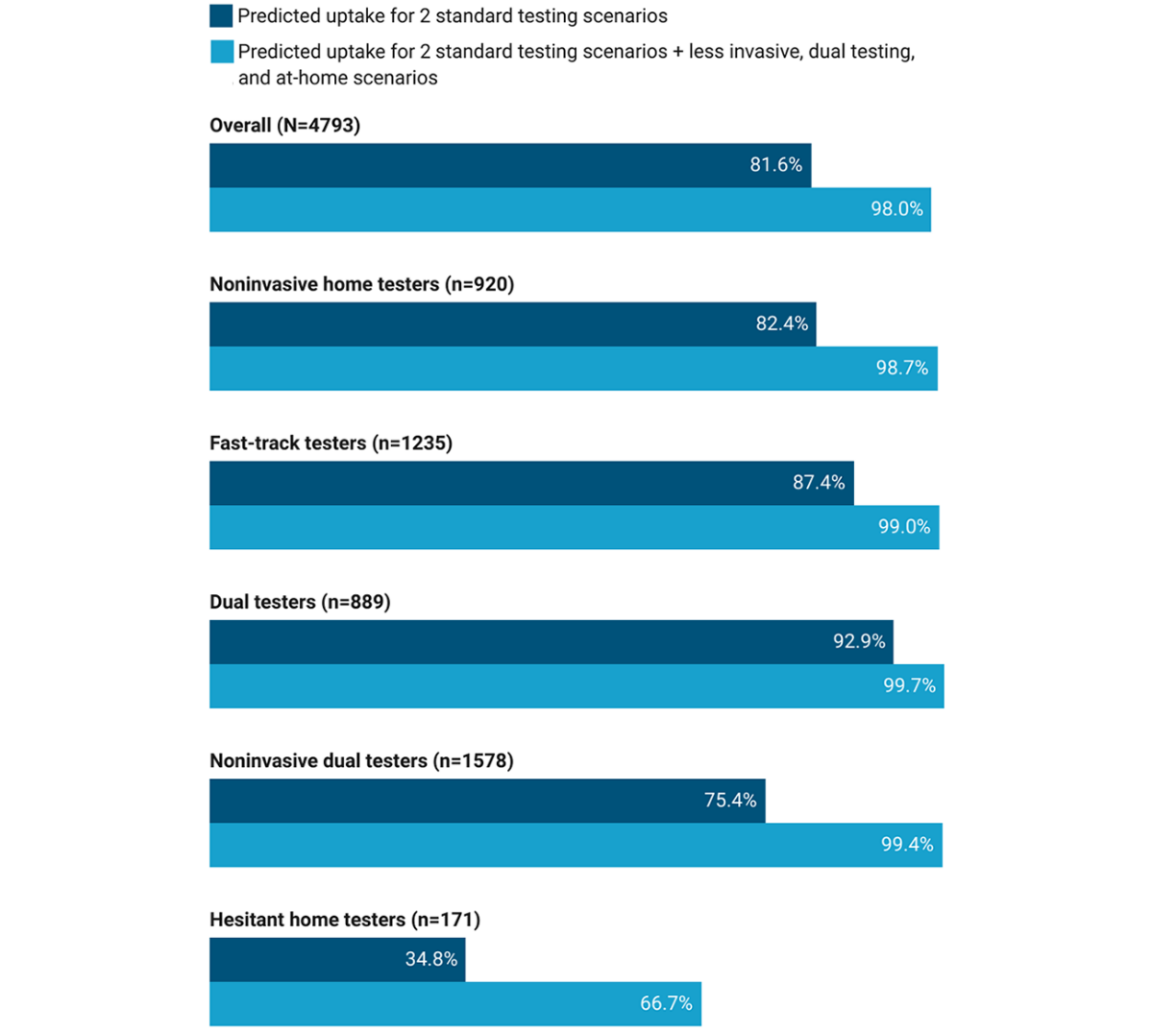
Simulated uptake of SARS-CoV-2 testing for 2 standard testing scenarios versus the addition of less invasive dual testing and at-home scenarios, overall and by preference pattern.

Predicted testing uptake for the 2 standard scenarios among all participants was 81.6%, ranging from 34.8% for hesitant home testers to 92.9% for dual testers (Figure 2). By including less invasive testing, dual testing, and at-home testing scenarios in our second simulation, predicted testing uptake among all participants increased by 16.4 percentage points to 98.0%. The addition of these 3 scenarios had the biggest impact on hesitant home testers, with an increase in uptake from 34.8% to 66.7% (31.9 percentage points), and noninvasive dual testers, with an increase in uptake from 75.4% to 99.4% (24.0 percentage points).

In our simulation of all 6 scenarios (Table S2, Multimedia Appendix 6), the standard testing scenarios generally had the lowest predicted uptake, though with higher uptake for the drive-through option (overall 6.6%) compared with the walk-in community site option (overall 0.9%). Of the 3 additional scenarios, the dual testing scenario combining polymerase chain reaction and serology had the highest predicted uptake overall (61.8%) and was highest for fast-track testers (60.8%), dual testers (65.0%), and noninvasive dual testers (80.9%). The at-home testing scenario had the highest predicted uptake for noninvasive home testers (37.9%) and hesitant home testers (38.2%); however, for noninvasive home testers, there was also similar uptake for the dual testing scenario (35.5%). For hesitant home testers, one-third (33.3%) were predicted to opt out of testing altogether.

### Demographic Characteristics of Participants by Preference Pattern

There were statistically significant differences by preference pattern in age group, gender, race/ethnicity, education, and employment, but not in geographic region, urbanicity, or presence of any comorbidity (Table S3, Multimedia Appendix 6). Hesitant home testers were older with less representation in the youngest age group of 18 to 39 years (69/171, 40.4%) compared with participants in other patterns (range 49.3%-56.1%). Fast-track testers were less often female (583/1235, 47.2%), especially when compared with hesitant home testers (100/171, 58.5%) and to a lesser extent when compared with participants in other patterns (range 50.5%-53.6%). Dual testers (625/889, 70.3%) and noninvasive dual testers (1059/1578, 67.1%) were more often non-Hispanic White, especially compared with hesitant home testers (85/171, 49.7%) and to a lesser extent with noninvasive home testers (516/920, 56.1%) and fast-track testers (725/1235, 58.6%). Dual testers tended to be college graduates (628/889, 70.6%), especially compared with noninvasive home testers (508/920, 55.2%) and to a lesser extent when compared with participants in other patterns (range 59.1%-64.3%). Regarding employment, hesitant home testers were more often out of work (37/171, 21.6%) compared with participants in other patterns (range 9.3%-13.8%).

### Previous SARS-CoV-2 Testing, COVID-19 Diagnosis, and Infection Concern by Preference Pattern

There were statistically significant differences by preference pattern for previous SARS-CoV-2 testing, reported COVID-19 diagnosis, concern about getting infected, concern about loved ones getting infected, concern about hospitals being overwhelmed, personally knowing someone who had died from COVID-19, and submitting a dried blood spot (DBS) for testing as part of our cohort study (Table S3, Multimedia Appendix 6). Fast-track testers (424/1235, 34.3%) and dual testers (298/889, 33.5%) more often had previously tested for SARS-CoV-2 compared with participants in other patterns (range 23.4%-25.9%). Reporting a previous laboratory-confirmed diagnosis of COVID-19 was lowest among dual testers (35/889, 3.9%) and noninvasive dual testers (51/1578, 3.2%) compared with participants in other patterns (range 5.3%-7.5%). Among those who did not report being previously diagnosed with COVID-19, dual testers were less often not at all worried or not too worried about getting infected with SARS-CoV-2 (179/889, 21.0%), especially when compared with hesitant home testers (52/171, 32.1%) and to a lesser extent participants in other patterns (range 23.9%-29.6%). A similar pattern was observed for concern about loved ones getting infected with SARS-CoV-2, where dual testers had the lowest proportion of people who reported being not at all worried or not too worried about loved ones getting infected (82/889, 9.2%) compared with hesitant home testers (38/171, 22.2%) and to a lesser extent compared with participants in other patterns (range 13.0%-17.5%). Likewise, dual testers had the lowest proportion of people who reported being not at all worried or not too worried about hospitals being overwhelmed by COVID-19 (112/889, 12.6%) compared to participants in other patterns (range 19.3%-32.2%).

Fast-track testers were more likely to personally know someone who had died from COVID-19 (320/1235, 25.9%) than hesitant home testers (34/171, 19.9%) and participants in other patterns (range 21.0%-22.5%). Noninvasive dual testers (1323/1578, 83.8%) and dual testers (741/889, 83.4%) were more likely to have submitted at least one DBS specimen for serology as part of our cohort study, compared with hesitant home testers (121/171, 70.8%), noninvasive home testers (671/920, 72.9%), and fast-track testers (915/1235, 74.1%).

## Discussion

### Principal Results

A one-size-fits-all approach to SARS-CoV-2 testing may alienate or exclude segments of the population with preferences for different testing modalities. If the goal is to increase and maintain testing uptake and engagement, then the identification of patterns of heterogeneous testing preferences could inform the design and implementation of complementary testing services that support greater coverage.

We identified substantial differences in preferences for aspects of SARS-CoV-2 testing, as shown by the differences in attribute relative importance and part-worth utilities. Overall, participants preferred getting both antibody and viral tests with less invasive specimens and fast turnaround time for results; however, the degree to which these features influenced participants’ choices varied across patterns. Our 6-scenario simulation showed that offering additional venues and test type options would increase testing uptake at a time when case numbers were increasing in many parts of the United States, with dual viral and antibody testing expected to have the biggest uptake. Though identifying previous infections via antibody tests does not help control transmission, including antibody tests with diagnostic testing could incentivize some people to get tested. An at-home testing option would also be expected to increase uptake, especially for participants in the noninvasive home testers and hesitant home testers patterns, which comprised about one-fifth of the sample.

Since this study was undertaken, there have been developments in SARS-CoV-2 viral and serologic testing that could address many of the distinct preferences across patterns, including the expansion of at-home specimen collection, affordable fully at-home tests commercially available without a prescription, rapid point-of-care tests, and less invasive specimens [41-44]. Though not yet approved by the US Food and Drug Administration, a promising saliva-based antibody test is in development [45], which may, in the future, allow for a single saliva specimen to be used for both serology and molecular testing. In our study, noninvasive home testers and hesitant home testers, who placed most importance on specimen type and venue, respectively, were more often non-Hispanic Black and Hispanic. These testing developments may provide a pathway to increase lower testing rates among Black and Latino individuals, who have experienced a disproportionate burden of cases, hospitalization, and deaths due to COVID-19 [46].

We also observed differences in sample characteristics by preference pattern, including demographic characteristics, previous testing, and COVID-19 diagnosis, and concern about infection. These differences could be leveraged to promote testing through media tools and campaigns targeting specific populations, similar to “The Conversation: Between Us, About Us” [47], created by the Kaiser Family Foundation’s Greater than COVID and the Black Coalition Against COVID and designed to address “some of the most common questions and concerns Black people have about COVID-19 vaccines,” or targeting specific behaviors, such as New York City’s Test and Trace Corps’ “Do it for them. Get Tested for COVID-19” advertisements on Twitter, bus shelters, and pizza boxes [48-50] with pictures of families of different races and ethnicities, sometimes multigenerational.

Across most of the preference patterns, the large negative utilities that we observed for the no-test option indicated a willingness to test. The exception was the hesitant home testers pattern, which had a positive utility for the none option, suggesting that this group of participants would be more likely to opt out of testing altogether compared to participants in the other patterns. Though hesitant home testers were least prevalent in our study, qualitative work may be warranted to understand the factors that influence their willingness to test.

### Limitations

Our results should be interpreted in the context of their limitations. An important limitation of our analysis is related to latent class analysis in general, as best practices for using it to study heterogeneity in preferences in health-related research are still evolving [15]. We selected 5 classes after comparing sample size, fit statistics, and overall interpretability of 2 to 10 classes. On the one hand, the hesitant home testers pattern was small relative to the other patterns, and one could argue that it could have been combined with a larger class in a solution with fewer groups. However, in every lower dimension solution, a similarly small-sized class was identified that was strongly influenced by venue and specimen type, and had a nonnegative utility for opting out of testing. On the other hand, it is possible that additional distinct patterns of preference remained undetected with only 5 classes.

Another potential limitation is that the stated preferences regarding SARS-CoV-2 testing in our DCE may not necessarily align with actual behavior (ie, revealed preferences); however, a systematic review and meta-analysis found that, in general, stated preferences in DCEs did align with revealed preferences [10]. To minimize cognitive burden, DCE design must balance the inclusion of relevant and actionable attributes and levels with the complexity of each choice task [51]. However, one reason for lack of concordance between stated and revealed preferences in general is the omission of attributes in DCEs that may influence real-life decisions [10,29]. Aspects of accessibility including cost, transportation time, availability of testing, and wait time could be explored in future studies, as well as how participants’ prior knowledge of test options may have influenced their decisions, the effects of operator error, and test validity (ie, sensitivity and specificity). Nevertheless, the different patterns of preferences for features of the test and testing experience as ascertained in our study could be used to inform the development of strategies deployed by public health agencies, who can account for the operating characteristics of tests. In some instances, even a less accurate test implemented at scale could have a larger public health impact than a more accurate test with lower uptake [52].

Although our sample was large and geographically diverse, it was not a nationally representative sample, so it may be that there are additional patterns of preferences that exist beyond our study in other populations. Not all testing options are available in every jurisdiction, and different patterns of testing preferences may emerge in different settings. Furthermore, most participants (78.3%) in the DCE had already completed at-home self-collection of a DBS specimen as part of our larger cohort study, which may have influenced preferences regarding the venue of testing.

Lastly, participants’ preferences about SARS-CoV-2 testing may change over time as the pandemic continues to evolve. Research on other topics has demonstrated that choices stated in a DCE are generally consistent, with good test-retest reliability [53,54]; however, knowledge about SARS-CoV-2 and COVID-19 has rapidly evolved and is widely disseminated in mainstream media [55], which could plausibly impact preferences. The first report of reinfection and the potential waning of antibodies appeared in the United States in October 2020 [56], approximately 1 month after the completion of our DCE, and could influence current preferences about antibody testing. In addition, the availability of highly efficacious vaccines starting in December 2020 [57] could have an impact on testing service preferences more globally, potentially causing more people to opt out of testing when case numbers, hospitalizations, and deaths decrease. It will also be important to examine preferences since new testing modalities have become available, such as fully at-home molecular tests that provide rapid results [58-60], and as vaccine uptake increases.

### Conclusions

Our study may inform ways to better design and deliver SARS-CoV-2 testing services in line with pandemic response goals. The heterogeneity in preferences observed across patterns highlights that having more options available (and educating the public about their availability) is one way to increase testing uptake in an emerging and ongoing pandemic. Importantly, our analysis highlights that preferences for SARS-CoV-2 testing differ by population characteristics, including demographics, which must also be considered in the context of existing health disparities in the United States. Even as increasing proportions of the population are vaccinated, we anticipate that testing will remain a critical tool in the pandemic response until vaccine coverage and herd immunity are sufficiently high to reduce transmission and control more pathogenic or virulent variants; offering a mix of testing options is an important aspect of increasing and maintaining testing uptake.

## Appendix

Multimedia Appendix 1 Desktop example of SARS-CoV-2 testing preferences choice task.

Multimedia Appendix 2 SARS-CoV-2 testing discrete choice experiment attributes and levels.

Multimedia Appendix 3 Definition of variables from the CHASING COVID Cohort Study.

Multimedia Appendix 4 Respondent quality details.

Multimedia Appendix 5 Summary of best replications of latent class analysis multinomial logit.

Multimedia Appendix 6 Supplementary tables on zero-centered part-worth utilities for SARS-CoV-2 testing attribute levels by preference pattern (Table S1); simulated uptake of standard testing, less invasive testing, dual testing, and at-home testing scenarios by preference pattern (Table S2); and sample characteristics, previous testing, COVID-19 diagnosis, and concern about infection stratified by preference pattern for SARS-CoV-2 testing (Table S3).

Multimedia Appendix 7 CHERRIES (Checklist for Reporting Results of Internet E-Surveys) checklist.

## Abbreviations

CHASING COVID Cohort Study: Communities, Households, and SARS-CoV-2 Epidemiology Cohort Study
CUNY: City University of New York
DBS: dried blood spot
DCE: discrete choice experiment
MNL: multinomial logit
NIH: National Institutes of Health
NP: nasopharyngeal
RFC: Randomized First Choice
