# Supplementary material for: Patterns of SARS-CoV-2 Testing Preferences in a National Cohort in the United States: Latent Class Analysis of a Discrete Choice Experiment
Source: JMIR Public Health Surveill. 2021 Dec 30;7(12):e32846. doi: 10.2196/32846 (PMC8722498; doi:10.2196/32846)
Supplement: Multimedia Appendix 1 [file publichealth_v7i12e32846_app1.pdf]

Multimedia Appendix 1. Desktop example of SARS-CoV-2 testing preferences choice task

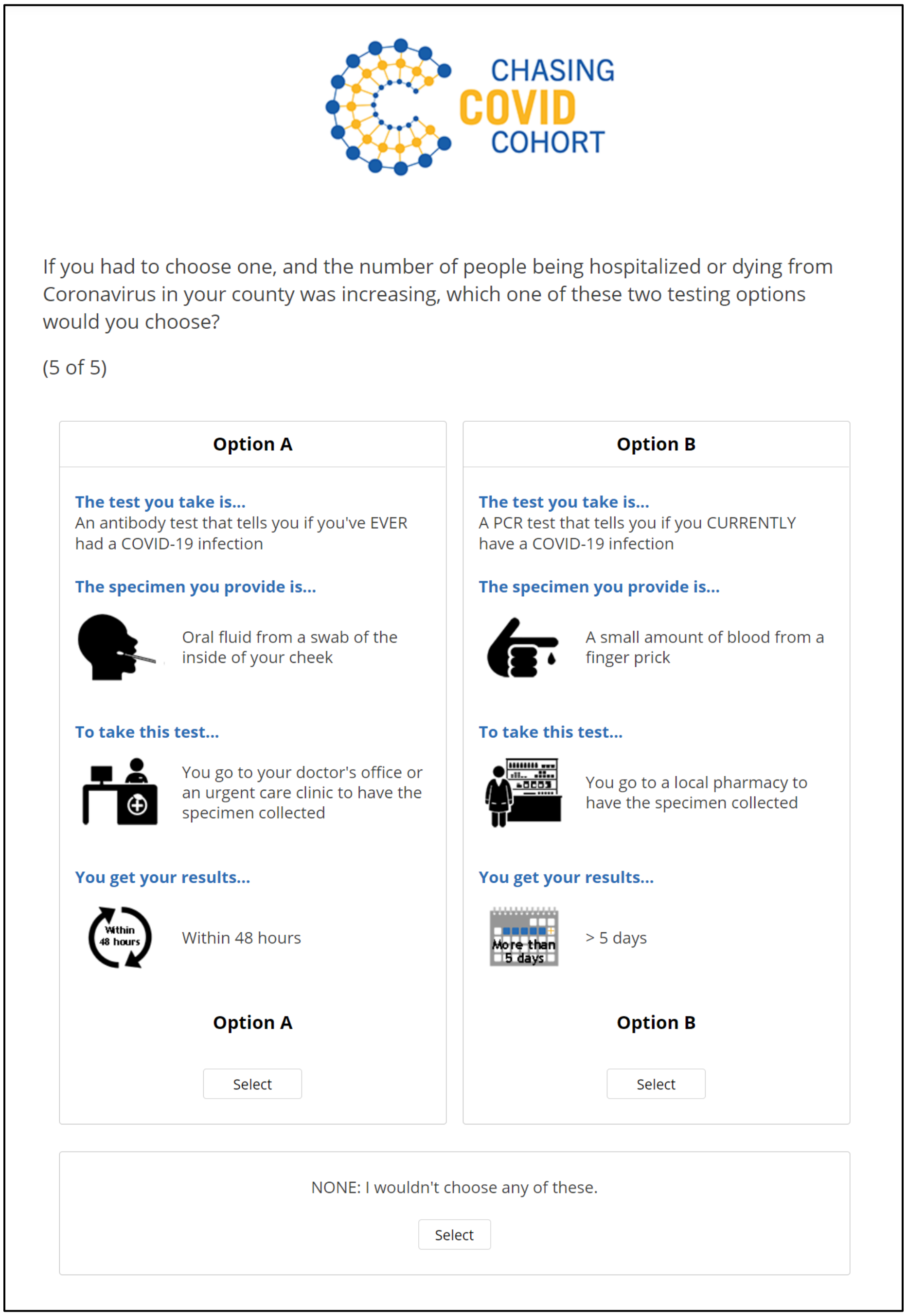

Desktop example of SARS-CoV-2 testing preferences choice task. Designed and implemented using Sawtooth Lighthouse Studio 9.8.1 (Sawtooth Software, Provo, UT). Images from The Noun Project. Additional information on the design of the survey can be found at:

Zimba R, Kulkarni S, Berry A, You W, Mirzayi C, Westmoreland D, Parcesepe A, Waldron L, Rane M, Kochhar S, Robertson M, Maroko A, Grov C, Nash D. SARS-CoV-2 Testing Service Preferences of Adults in the United States: Discrete Choice Experiment. JMIR Public Health Surveill. 2020;6:e25546. doi:10.2196/25546. PMID: 33315584
