# Supplementary material for: Patterns of SARS-CoV-2 Testing Preferences in a National Cohort in the United States: Latent Class Analysis of a Discrete Choice Experiment
Source: JMIR Public Health Surveill. 2021 Dec 30;7(12):e32846. doi: 10.2196/32846 (PMC8722498; doi:10.2196/32846)
Supplement: Multimedia Appendix 2 [file publichealth_v7i12e32846_app2.pdf]

Multimedia Appendix 2. SARS-CoV-2 testing discrete choice experiment attributes and levels

Attributes & levels (abbreviated)[a]	Descriptive level text[b]
**Test**	
Serology	An antibody test that tells you if you've EVER had a COVID-19 infection
PCR[c]	A PCR test that tells you if you CURRENTLY have a COVID-19 infection
Both tests	BOTH an antibody test (EVER infected) and a PCR test (CURRENTLY infected)
**Specimen type**	
Finger prick	A small amount of blood from a finger prick
Blood draw	A small tube of blood taken from your arm
Cheek	Oral fluid from a swab of the inside of your cheek
Spit	A spit sample collected in a small cup
Nasal shallow	A SHALLOW swab of the inside of your nostrils
NP[d] swab	A DEEP swab that goes far into your nasal passages
Urine	A urine sample collected in a small cup
**Venue**	
Home collection, receiving & returning kit in mail	You are mailed a package with the test kit, you collect the specimen, and mail it back to the lab
Home collection, receiving kit in mail & returning to a collection site	You are mailed a package with the test kit, you collect the specimen, and drop it off at a collection site near your home
Doctor’s office or urgent care clinic	You go to your doctor's office or an urgent care clinic to have the specimen collected
Walk-in community testing site	You go to a walk-in community testing site to have the specimen collected
Drive-through community testing site	You go to a drive-thru community testing site to have the specimen collected (you stay in your car)
Pharmacy	You go to a local pharmacy to have the specimen collected
**Results turnaround time**	
Immediate	Immediately (within 15 minutes)
Same day	On the same day
48 hours	Within 48 hours
5 days	Within 5 days
Greater than 5 days	> 5 days
**None – I wouldn’t choose any of these**	

[a]Some combinations of attribute levels were prohibited. For example, a test scenario that included a specimen collected at home and returned to the lab via mail could not also include the immediate test result level.
[b]Descriptive text was displayed in the choice exercise.
[c]PCR=polymerase chain reaction
[d]NP=nasopharyngeal
