# Supplementary material for: Patterns of SARS-CoV-2 Testing Preferences in a National Cohort in the United States: Latent Class Analysis of a Discrete Choice Experiment
Source: JMIR Public Health Surveill. 2021 Dec 30;7(12):e32846. doi: 10.2196/32846 (PMC8722498; doi:10.2196/32846)
Supplement: Multimedia Appendix 3 [file publichealth_v7i12e32846_app3.pdf]

Multimedia Appendix 3. Definition of variables from the CHASING COVID Cohort

For this analysis, age was categorized into 3 groups (18-39 years, 40-59 years, and ≥60 years). Gender was recategorized into three groups (male, female, transgender/non-binary/other); Race and ethnicity were ascertained by two questions related to Hispanic heritage and race, and was categorized into five groups (Hispanic, non-Hispanic Black, Asian/Pacific Islander, non-Hispanic white, and other). Education was ascertained by asking about highest grade or year of school completed (less than high school diploma, grade 12 or GED [high school graduate], college 1-3 years [some college or technical school], college 4 years or more [college graduate]). Region was ascertained based on U.S. state or territory of residence and categorized into 4 census regions (Northeast, Midwest, South, West) and Puerto Rico. Urbanicity was categorized based on city locale assignments from the National Center for Education Statistics using ZIP Code Tabulation Areas with urban defined as living in an urbanized area and inside a principal city [1].

Having any comorbidities (yes/no) was based on a question about ever being told by a healthcare professional that the participant had any chronic conditions in a list (i.e., coronary artery disease, diabetes, hypertension, cancer, asthma, chronic obstructive pulmonary disease, kidney disease, HIV/AIDS, immunosuppression, and depression). Employment was ascertained in each survey by asking about the participant’s current situation and was recategorized as employed (employed for wages, self-employed), out of work, or not in the workforce (homemaker, student, retired). Participants were asked in baseline and follow-up surveys about past SARS-CoV-2 testing and frequency of testing, which we recategorized as never (inclusive of don’t know/not sure) or yes (one or more times). Participants were also asked in the baseline

and follow-up surveys if they had previously received a laboratory-confirmed diagnosis of COVID-19.

For concern about infection, participants were asked separate questions about the intensity of their concern about themselves or loved ones getting sick from SARS-CoV-2, and about hospitals in their area being overwhelmed by COVID-19 cases, with responses recategorized as not at all worried/not too worried, somewhat worried, and very worried. The question regarding concern about themselves getting infected was restricted to participants who reported not having a previous laboratory-confirmed diagnosis of COVID-19. At each survey participants were asked whether they personally knew anyone who had died from COVID-19 since the previous survey, which was combined into a cumulative variable.

Finally, participants were asked to submit a dried blood spot (DBS) specimen for serology testing at the first follow-up survey which remained open from April 2020-July 2020. DBS specimens were tested using the Bio-Rad Platelia SRAS-CoV-2 Total Antibody Assay [2,3]. Participants were defined as seropositive if their specimen was reactive.

References

1. Geverdt D. ZIP Code Tabulation Area (ZCTA) Locale Assignments File Documentation, 2017. :9.

2. SARS-CoV-2 / COVID-19 Antibody Detection [Internet]. Bio-Rad Lab. [cited 2021 May 26]. Available from: /featured/en/sars-cov-2-surveillance.html

3. Health C for D and R. EUA Authorized Serology Test Performance. FDA [Internet] FDA; 2021 May 25 [cited 2021 May 26]; Available from: https://www.fda.gov/medical-devices/coronavirus-disease-2019-covid-19-emergency-use-authorizations-medical-devices/eua-authorized-serology-test-performance
