# Supplementary material for: Patterns of SARS-CoV-2 Testing Preferences in a National Cohort in the United States: Latent Class Analysis of a Discrete Choice Experiment
Source: JMIR Public Health Surveill. 2021 Dec 30;7(12):e32846. doi: 10.2196/32846 (PMC8722498; doi:10.2196/32846)
Supplement: Multimedia Appendix 4 [file publichealth_v7i12e32846_app4.pdf]

Multimedia Appendix 4.

Respondent quality details

A total of 392 participants exhibited straightlining choice behavior. The median response time for the DCE tasks for all 4,793 participants was 144 seconds, or about 29 seconds per task. 473 participants had completion times in the 10th percentile, which was 76 seconds for all tasks or about 15 seconds per task; 239 participants had completion times in the 5th percentile, which was 58 seconds for all tasks, or about 12 seconds per task. The analysis excluding participants who exhibited straightlining and 5th percentile speed included 4,765 participants; the analysis excluding participants who exhibited straightlining and 10th percentile speed included 4,746 participants; the analysis excluding participants who exhibited straightlining or 5th percentile speed included 4,190 participants; and the analysis excluding participants who exhibited straightlining or 10th percentile speed included 3,975 participants.
