# Supplementary material for: Patterns of SARS-CoV-2 Testing Preferences in a National Cohort in the United States: Latent Class Analysis of a Discrete Choice Experiment
Source: JMIR Public Health Surveill. 2021 Dec 30;7(12):e32846. doi: 10.2196/32846 (PMC8722498; doi:10.2196/32846)
Supplement: Multimedia Appendix 5 [file publichealth_v7i12e32846_app5.pdf]

Multimedia Appendix 5. Summary of best replications of latent class analysis multinomial logit[a]

Groups	Replication	LL[b]	LL difference from previous solution	AIC[c]	AIC difference from previous solution	BIC[d]	BIC difference from previous solution	Min class size	Max class size	Average class size
2	5	-16284	N/A	32642	N/A	32941	N/A	308	4485	2397
3	2	-15975	309	32062	580	32515	427	193	2421	1598
4	2	-15768	207	31686	376	32292	223	162	2252	1198
5	2	-15602	165	31393	293	32153	139	171	1578	959
6	1	-15549	53	31325	68	32238	-85	180	1521	799
7	4	-15504	46	31271	53	32338	-100	175	1612	685
8	3	-15477	27	31256	15	32477	-138	142	920	599
9	5	-15431	46	31202	53	32577	-100	150	1116	533
10	1	-15404	27	31186	16	32714	-137	116	1339	479

[a]Starting seed=1

[b]LL=Log-likelihood

[c]AIC=Akaike information criterion

[d]BIC=Bayesian information criterion
