# Supplementary material for: Patterns of SARS-CoV-2 Testing Preferences in a National Cohort in the United States: Latent Class Analysis of a Discrete Choice Experiment
Source: JMIR Public Health Surveill. 2021 Dec 30;7(12):e32846. doi: 10.2196/32846 (PMC8722498; doi:10.2196/32846)
Supplement: Multimedia Appendix 6 [file publichealth_v7i12e32846_app6.pdf]

Multimedia Appendix 6

Table S1. Zero-centered part-worth utilities[a] for SARS-CoV-2 testing attribute levels by preference pattern

	Overall 4793 (100%)	Noninvasive home testers 920 (19.2%)	Fast-track testers 1235 (25.8%)	Dual testers 889 (18.5%)	Noninvasive dual testers 1578 (32.9%)	Hesitant home testers 171 (3.6%)
	Mean (95% CI)	Mean (95% CI)	Mean (95% CI)	Mean (95% CI)	Mean (95% CI)	Mean (95% CI)
**Test type**						
Serology	-41.7 (-42.6, -40.9)	-28.2 (-29.1, -27.2)	-34.5 (-35.4, -33.5)	-97 (-98, -96)	-26.5 (-27.1, -25.9)	-20 (-20.7, -19.3)
PCR	-22.2 (-22.8, -21.6)	-11.4 (-12.2, -10.6)	-18.5 (-19, -18)	3.7 (3, 4.3)	-46.8 (-47.2, -46.4)	-16.1 (-16.7, -15.6)
Both tests	64 (63.3, 64.6)	39.5 (38.5, 40.6)	53 (52.3, 53.6)	93.3 (92.8, 93.8)	73.3 (73, 73.7)	36.1 (35.4, 36.9)
**Specimen type**						
Finger prick	19.3 (19, 19.5)	23.4 (23.1, 23.7)	19.7 (19.4, 20)	2 (1.6, 2.3)	25.7 (25.6, 25.9)	24.5 (24.3, 24.7)
Blood draw	-18 (-18.5, -17.6)	-41.3 (-42.1, -40.4)	-20.7 (-21, -20.4)	-18.7 (-19, -18.5)	-1.7 (-2, -1.4)	-21.1 (-21.6, -20.5)
Cheek swab	29.5 (29.2, 29.7)	34 (33.7, 34.3)	25.4 (25.1, 25.6)	16.4 (16.1, 16.7)	37.8 (37.7, 38)	25.6 (25.3, 25.9)
Spit sample	25.3 (24.9, 25.7)	48.6 (47.9, 49.3)	13.3 (13, 13.6)	14.2 (13.9, 14.5)	28.1 (27.9, 28.4)	18.7 (18, 19.3)
Shallow nasal swab	1.4 (1.1, 1.7)	9.4 (9, 9.8)	7.5 (7.2, 7.7)	6.8 (6.6, 7)	-10.5 (-10.7, -10.3)	-3.5 (-3.9, -3.2)
Nasopharyngeal swab	-72.5 (-73.4, -71.6)	-92.1 (-93, -91.1)	-51.1 (-52, -50.3)	-30.5 (-31.4, -29.5)	-100.9 (-101.4, -100.3)	-77.7 (-78.4, -76.9)
Urine	15 (14.8, 15.3)	17.9 (17.7, 18.2)	6 (5.8, 6.3)	9.9 (9.7, 10)	21.3 (21.2, 21.4)	33.5 (33, 34.1)
**Venue**						
Home collection, receiving kit in mail & returning kit in mail	17.4 (16.6, 18.2)	55.3 (54, 56.6)	5.4 (5, 5.8)	-4.1 (-4.5, -3.7)	8.6 (8.3, 8.9)	93.2 (91.3, 95.2)
Home collection, receiving kit in the mail & returning to a collection site	12.6 (12, 13.3)	42.7 (41.7, 43.7)	-8.8 (-9.2, -8.3)	-5.2 (-5.6, -4.8)	16.8 (16.5, 17.1)	60.2 (58.9, 61.6)
Doctor's office or urgent care clinic	-16.1 (-16.5, -15.6)	-41.6 (-42.4, -40.8)	-5.4 (-5.6, -5.1)	-4 (-4.3, -3.7)	-13.2 (-13.4, -13)	-44.6 (-45.4, -43.8)
Walk-in community testing site	-18.9 (-19.4, -18.3)	-44.5 (-45.4, -43.6)	-6.6 (-6.9, -6.3)	-3.8 (-4.1, -3.5)	-16 (-16.2, -15.7)	-75.9 (-77.4, -74.4)
Drive through community testing site	4.2 (3.9, 4.4)	2.7 (2.5, 2.9)	10.8 (10.6, 11)	12.2 (12, 12.4)	-3.5 (-3.7, -3.4)	-6.9 (-7.3, -6.5)
Pharmacy	0.8 (0.5, 1.1)	-14.5 (-15.1, -14)	4.5 (4.3, 4.6)	4.9 (4.8, 5)	7.3 (7.2, 7.4)	-26.1 (-26.8, -25.3)
**Results turnaround time**						
Immediate	64.7 (64, 65.3)	42 (41.1, 43)	98.6 (97.9, 99.2)	64.9 (64.5, 65.4)	54.7 (54.2, 55.1)	32.5 (31.1, 33.8)

	Overall<br>4793 (100%)	Noninvasive home testers<br>920 (19.2%)	Fast-track testers<br>1235 (25.8%)	Dual testers<br>889 (18.5%)	Noninvasive dual testers<br>1578 (32.9%)	Hesitant home testers<br>171 (3.6%)
	**Mean (95% CI)**	**Mean (95% CI)**	**Mean (95% CI)**	**Mean (95% CI)**	**Mean (95% CI)**	**Mean (95% CI)**
Same day	38.8 (38.3, 39.3)	30.4 (29.8, 30.9)	63.8 (63.3, 64.4)	40.5 (40.2, 40.9)	25.1 (24.7, 25.5)	21.2 (20.3, 22.1)
48 hours	8.3 (8.1, 8.4)	1.4 (1.2, 1.7)	13.6 (13.5, 13.7)	8.4 (8.3, 8.5)	9.5 (9.5, 9.6)	-5.2 (-5.6, -4.8)
5 days	-43 (-43.5, -42.5)	-26.2 (-26.9, -25.5)	-66.9 (-67.4, -66.5)	-39 (-39.4, -38.6)	-39 (-39.3, -38.8)	-18.5 (-19.5, -17.4)
Greater than 5 days	-68.7 (-69.6, -67.9)	-47.7 (-48.6, -46.7)	-109.1 (-109.9, -108.3)	-74.9 (-75.4, -74.3)	-50.2 (-50.8, -49.6)	-30 (-31.6, -28.3)
**None**	-232.6 (-234.4, -230.8)	-299.6 (-302, -297.2)	-238 (-238.9, -237.1)	-217.4 (-218.3, -216.4)	-226.6 (-227.3, -225.9)	32.5 (23.8, 41.1)

[a]Direction of the utility values implies the preference for the attribute level (negative=less preferred; positive=more preferred) and the magnitude of the utility implies the strength of preference.

[b]CI=Confidence interval

[c]PCR=polymerase chain reaction

Table S2. Simulated uptake[a] of standard testing, less invasive testing, dual testing, and at-home testing scenarios by preference pattern

	Overall 4793 (100%)	Noninvasive home testers 920 (19.2%)	Fast-track testers 1235 (25.8%)	Dual testers 889 (18.5%)	Noninvasive dual testers 1578 (32.9%)	Hesitant home testers 171 (3.6%)
	% (95% CI[b])	% (95% CI)	% (95% CI)	% (95% CI)	% (95% CI)	% (95% CI)
Standard testing scenarios						
Viral test, NP[c] swab, drive-through community testing site, results within 48 hours	6.6% (6.4%, 6.7%)	6.6% (6.3%, 6.8%)	10.4% (10.1%, 10.7%)	10.9% (10.6%, 11.2%)	1.3% (1.3%, 1.4%)	4.3% (3.9%, 4.6%)
Viral test, NP swab, walk-in community testing site, results within 48 hours	0.9 (0.9%, 1%)	0.5% (0.5%, 0.6%)	1.8% (1.7%, 1.9%)	1.7% (1.6%, 1.8%)	0.1% (0.1%, 0.1%)	0.3% (0.2%, 0.4%)
Less invasive testing scenario						
Viral test, saliva, walk-in community testing site, results within 48 hours	12.7% (12.6%, 12.9%)	18.2% (17.9%, 18.5%)	15.2% (14.9%, 15.4%)	11.8% (11.6%, 12.1%)	8.9% (8.7%, 9.2%)	5.8% (5.3%, 6.3%)
Dual testing scenario						
Viral and antibody tests, finger prick, walk-in community testing site, results within 48 hours	61.8% (61.2%, 62.4%)	35.5% (34.6%, 36.4%)	60.8% (60.3%, 61.3%)	65% (64.5%, 65.5%)	80.9% (80.5%, 81.3%)	18.3% (17%, 19.5%)
At-home testing scenario						
Viral test, shallow nasal swab, home collection, receiving kit in mail & returning kit in mail, results within 5 days	16% (15.6%, 16.4%)	37.9% (37.1%, 38.7%)	10.9% (10.6%, 11.1%)	10.3% (10%, 10.6%)	8.1% (7.9%, 8.3%)	38.2% (37.2%, 39.1%)
None	2% (1.8%, 2.1%)	1.3% (1.2%, 1.4%)	1% (0.9%, 1.1%)	0.3% (0.2%, 0.3%)	0.6% (0.6%, 0.7%)	33.3% (31.7%, 34.8%)

[a]Simulated preferences represent the proportion of individuals in each pattern that were predicted to prefer any given scenario. For each pattern, the lowest percentage=the least preferred and the highest percentage=the most preferred scenario.

[b]CI=confidence interval

[c]NP=nasopharyngeal

Table S3. Sample characteristics, previous testing, COVID-19 diagnosis, and concern about infection stratified by preference pattern for SARS-CoV-2 testing

	Overall 4793 (100%)	Noninvasive home testers 920 (19.2%)	Fast-track testers 1235 (25.8%)	Dual testers 889 (18.5%)	Noninvasive dual testers 1,78 (32.9%)	Hesitant home testers 171 (3.6%)	
	N (%)	N (%)	N (%)	N (%)	N (%)	N (%)	P-value
Age group							.002
18-39 years	2489 (51.9%)	516 (56.1%)	662 (53.6%)	464 (52.2%)	778 (49.3%)	69 (40.4%)	
40-59 years	1502 (31.3%)	272 (29.6%)	378 (30.6%)	280 (31.5%)	504 (31.9%)	68 (39.8%)	
>=60 years	802 (16.7%)	132 (14.3%)	195 (15.8%)	145 (16.3%)	296 (18.8%)	34 (19.9%)	
Gender							.012
Female	2468 (51.5%)	465 (50.5%)	583 (47.2%)	474 (53.3%)	846 (53.6%)	100 (58.5%)	
Male	2193 (45.8%)	432 (47%)	619 (50.1%)	385 (43.3%)	691 (43.8%)	66 (38.6%)	
Transgender, non-binary, other	132 (2.8%)	23 (2.5%)	33 (2.7%)	30 (3.4%)	41 (2.6%)	5 (2.9%)	
Race/ethnicity							<.001
Asian/Pacific Islander	356 (7.4%)	66 (7.2%)	96 (7.8%)	63 (7.1%)	118 (7.5%)	13 (7.6%)	
Hispanic	788 (16.4%)	179 (19.5%)	239 (19.4%)	117 (13.2%)	218 (13.8%)	35 (20.5%)	
Non-Hispanic Black	471 (9.8%)	116 (12.6%)	128 (10.4%)	68 (7.6%)	134 (8.5%)	25 (14.6%)	
Non-Hispanic White	3009 (62.8%)	516 (56.1%)	724 (58.6%)	625 (70.3%)	1059 (67.1%)	85 (49.7%)	
Other	161 (3.4%)	40 (4.3%)	47 (3.8%)	15 (1.7%)	47 (3%)	12 (7%)	
Missing	8 (0.2%)	3 (0.3%)	1 (0.1%)	1 (0.1%)	2 (0.1%)	1 (0.6%)	
Education							<.001
Less than high school	69 (1.4%)	15 (1.6%)	17 (1.4%)	10 (1.1%)	25 (1.6%)	2 (1.2%)	
High school graduate	448 (9.3%)	120 (13%)	107 (8.7%)	64 (7.2%)	133 (8.4%)	24 (14%)	
Some college or technical school	1241 (25.9%)	275 (29.9%)	335 (27.1%)	184 (20.7%)	403 (25.5%)	44 (25.7%)	
College graduate	3027 (63.2%)	508 (55.2%)	775 (62.8%)	628 (70.6%)	1015 (64.3%)	101 (59.1%)	
Missing	8 (0.2%)	2 (0.2%)	1 (0.1%)	3 (0.3%)	2 (0.1%)	0 (0%)	
Employment							.002
Employed	3077 (64.2%)	597 (64.9%)	803 (65%)	579 (65.1%)	1003 (63.6%)	95 (55.6%)	
Out of work	605 (12.6%)	119 (12.9%)	171 (13.8%)	83 (9.3%)	195 (12.4%)	37 (21.6%)	

	Overall 4793 (100%)	Noninvasive home testers 920 (19.2%)	Fast-track testers 1235 (25.8%)	Dual testers 889 (18.5%)	Noninvasive dual testers 1,78 (32.9%)	Hesitant home testers 171 (3.6%)	
	N (%)	N (%)	N (%)	N (%)	N (%)	N (%)	P-value
Not in the workforce	1101 (23%)	201 (21.8%)	258 (20.9%)	226 (25.4%)	378 (24%)	38 (22.2%)	
Missing	10 (0.2%)	3 (0.3%)	3 (0.2%)	1 (0.1%)	2 (0.1%)	1 (0.6%)	
Region							.27
Northeast	1189 (24.8%)	205 (22.3%)	308 (24.9%)	244 (27.4%)	386 (24.5%)	46 (26.9%)	
Midwest	847 (17.7%)	145 (15.8%)	225 (18.2%)	159 (17.9%)	296 (18.8%)	22 (12.9%)	
South	1552 (32.4%)	310 (33.7%)	410 (33.2%)	280 (31.5%)	489 (31%)	63 (36.8%)	
West	1152 (24%)	245 (26.6%)	280 (22.7%)	199 (22.4%)	390 (24.7%)	38 (22.2%)	
US Dependent	7 (0.1%)	3 (0.3%)	1 (0.1%)	1 (0.1%)	2 (0.1%)	0 (0%)	
Missing	46 (1%)	12 (1.3%)	11 (0.9%)	6 (0.7%)	15 (1%)	2 (1.2%)	
Urban	2074 (43.3%)	373 (40.5%)	558 (45.2%)	398 (44.8%)	673 (42.6%)	72 (42.1%)	.22
Any comorbidity (baseline)	1895 (39.5%)	352 (38.3%)	490 (39.7%)	343 (38.6%)	633 (40.1%)	77 (45%)	.50
Reported previously testing for SARS-CoV-2	1378 (28.8%)	238 (25.9%)	424 (34.3%)	298 (33.5%)	378 (24%)	40 (23.4%)	<.001
Reported previously receiving a laboratory-confirmed diagnosis of COVID-19	243 (5.1%)	55 (6%)	93 (7.5%)	35 (3.9%)	51 (3.2%)	9 (5.3%)	<.001
Concern about getting infected with SARS-CoV-2 (among those without a previous COVID-19 diagnosis)							<.001
Not at all worried/not too worried	1190 (26.1%)	234 (27.1%)	273 (23.9%)	179 (21%)	452 (29.6%)	52 (32.1%)	
Somewhat worried	2278 (50.1%)	406 (46.9%)	573 (50.2%)	472 (55.3%)	759 (49.7%)	68 (42%)	
Very worried	1075 (23.6%)	223 (25.8%)	294 (25.7%)	202 (23.6%)	314 (20.6%)	42 (25.9%)	
Missing	7 (0.2%)	2 (0.2%)	2 (0.2%)	1 (0.1%)	2 (0.1%)	0 (0%)	
Concern about loved ones getting infected with SARS-CoV-2							<.001
Not at all worried/not too worried	695 (14.5%)	161 (17.5%)	160 (13%)	82 (9.2%)	254 (16.1%)	38 (22.2%)	
Somewhat worried	1968 (41.1%)	333 (36.2%)	524 (42.4%)	386 (43.4%)	660 (41.8%)	65 (38%)	

	**Overall 4793 (100%)**	**Noninvasive home testers 920 (19.2%)**	**Fast-track testers 1235 (25.8%)**	**Dual testers 889 (18.5%)**	**Noninvasive dual testers 1,78 (32.9%)**	**Hesitant home testers 171 (3.6%)**	**P-value**
	**N (%)**	**N (%)**	**N (%)**	**N (%)**	**N (%)**	**N (%)**	
Very worried	2121 (44.3%)	423 (46%)	549 (44.5%)	420 (47.2%)	662 (42%)	67 (39.2%)	
Missing	9 (0.2%)	3 (0.3%)	2 (0.2%)	1 (0.1%)	2 (0.1%)	1 (0.6%)	
Concern about hospitals getting overwhelmed by COVID-19							<.001
Not at all worried/not too worried	958 (20%)	205 (22.3%)	238 (19.3%)	112 (12.6%)	348 (22.1%)	55 (32.2%)	
Somewhat worried	2252 (47%)	410 (44.6%)	591 (47.9%)	445 (50.1%)	751 (47.6%)	55 (32.2%)	
Very worried	1574 (32.8%)	302 (32.8%)	404 (32.7%)	331 (37.2%)	477 (30.2%)	60 (35.1%)	
Missing	9 (0.2%)	3 (0.3%)	2 (0.2%)	1 (0.1%)	2 (0.1%)	1 (0.6%)	
Personally knew someone who had died from COVID-19	1083 (22.6%)	197 (21.4%)	320 (25.9%)	200 (22.5%)	332 (21%)	34 (19.9%)	.02
Submitted DBS for serology in the CHASING COVID Cohort study	3771 (78.7%)	671 (72.9%)	915 (74.1%)	741 (83.4%)	1323 (83.8%)	121 (70.8%)	<.001
DBS reactive	113 (3%)	18 (2.7%)	36 (3.9%)	18 (2.4%)	38 (2.9%)	3 (2.5%)	.41

[a]Among those who did not report previously receiving a COVID-19 diagnosis
[b]DBS=Dried blood spot
